# The prevalence of obesity in older adults in Iran: a systematic review and meta-analysis

**DOI:** 10.1186/s12877-019-1396-4

**Published:** 2019-12-23

**Authors:** Aliakbar Vaisi-Raygani, Masoud Mohammadi, Rostam Jalali, Akram Ghobadi, Nader Salari

**Affiliations:** Department of Nursing, School of Nursing and Midwifery, Kermanshah University of Medical Sciences, Kermanshah, Iran

**Keywords:** Obesity, BMI, Older adult, Iran, Meta-analysis

## Abstract

**Background:**

one of the most important age-dependent physiologic alterations in the body composition of older adult people is obesity and overweight, increasing the risk of cardiovascular disease and mortality rate.

**Objective:**

The aim of the present study is to determine the prevalence of obesity in older adults in Iran.

**Methods:**

The present study was conducted via meta-analysis and systematic review method, from March 2000 to October 2018. Subject-related literature was obtained via searches in ScienceDirect, Medline (PubMed), SID, Magiran, Scopus, and Google Scholar databases. Heterogeneity of studies was assessed using the I^2^ index, and data were analyzed by Comprehensive-Meta analysis software.

**Results:**

In the assessment of 18 studies and 29,943 persons aged over 50 years, the prevalence of obesity in older adults of Iran was 21.4% (95%CI: 26.6–16.9%) based on the meta-analysis. The highest obesity prevalence was obtained in older adults of Babol (Amir Shahr) which was 44.2% (95%CI: 41.1–47.2%) in 2007, while the minimum obesity prevalence was found in older adults of Razavi Khorasan which was 11.3% (95%CI, 10–12.8%) in 2007. Further, as the sample size and the study year increased, the obesity prevalence diminished in older Iranian adults (*p* < 0.05).

**Conclusion:**

This study suggests that the prevalence of obesity in the older adults of Iran is high. Accordingly, healthcare planners and politicians should consider effective and practical policies to reduce obesity in older adults.

## Background

Improved life conditions, reduced mortality and reproduction, and enhanced of lifetime and life expectancy have resulted in growing elderly population worldwide. According to WHO, this population will reach 12 billion persons in 2025, while the maximum growth of this population (approximately 70%) will be seen in developing countries [1].

In Iran, the population of older adult is also growing quickly, such that throughout the future 50 years, older adults would constitute 20% of the population [2]. Further, studies have shown that population over 60 years will be more than 9% of the total population of Iran by 2020 [1, 3].

As increase in the population of older adults could result in heightened risk of chronic diseases, disability, cognitive decline, increased need for social services and medicines, as well as related expenses, determining the factors affecting the life of older adults, as well as control and improvement of their quality could mitigate the difficulties of this human life stage [4]. Some of the most important age-dependent physiologic alterations in the body composition of older adults are elevated body fat mass, obesity, and overweight [5, 6]. In this regard, different studies have reported that the risk of cardiovascular disease and mortality rate among older adults increases in response to ageing as well as some factors such as obesity, lack of movement, diabetes, and hypertension [7–11].

In the studies performed in Iran and its different areas, different prevalence values have been reported including 11.3% in Razavi Khorasan [12], 33.1% in Yasooj [13], and 19.3% in Tehran [14].

As obesity rate shows inconsistency in different areas of Iran, and performing interventions for decreasing obesity prevalence in older adults needs exact and consistent information, and since total statistics of obesity prevalence in older adults of Iran are not comprehensive and clear, the question of this study is what the total prevalence of obesity is in the elderly population of Iran.

## Objectives

The aim of the present study is to determine the prevalence of obesity in older adults of Iran via systematic review and meta-analysis.

## Methods

The present study was performed using meta-analysis method and was the result of data extracted from studies performed on the prevalence of obesity in older adults (within the age range over 50 years and in healthy older adults without any comorbidities) of Iran [12–23].

The search strategy involved a series of complementary search methods including a comprehensive search of key bibliographic databases and manual search of reference lists or citation follow-ups of identified eligible articles and relevant reviews which would not be captured through the bibliographic databases search. Using relevant search terms developed from Medical Subject Headings (MeSH), a systematic search of internal and international journals was done along with searching in ScienceDirect, Medline (PubMed), SID, Magiran, Scopus, and Google Scholar databases from March 2000 to October 2018 (in studies in Iran, articles published before about 2000 contain outdated information and are not comparable in quality to more recent studies. Also, most of these studies were not available in search sources and the researchers failed to gain access to those resources. Thus, the authors restricted the study to beyond 2000 to keep the information up-to-date and available). The search terms to be used included the following: ‘BMI’, ‘obesity’, ‘Older adult’, ‘Body Mass Index’ both in Persian and English, and their possible combinations. In this study, obesity in older adults was assessed based on the body mass index where BMI ≥ 30 was considered as obesity [12–14].

Searching in Persian databases was performed using Persian keywords, while in English databases, it was via their equal English words. In Google Scholar database, searching was performed using both Persian and English keywords. Words “AND” and “OR” were employed in combinations to obtain more comprehensive articles, where “OR” was applied for different common names of a disorder such as “Older adult OR Aged”, “obesity OR body weight”, “BMI OR Body Mass Index”, while “AND” was utilized between words (BMI AND Obesity).

### Criteria for selection and evaluation of the literature

First, all related literature were collected using selected keywords and then, a list of article’s abstracts was prepared. After hiding the specifications of literature such as the journal’s name and the authors’ name, complete contents of articles were provided to reviewers. Each article was separately reviewed by two reviewers. If the article was rejected by them, they expressed the reason, and if there was any controversy between the reviewers, the article was reviewed by a third referee whose opinion was considered as the final decision. All Persian and English literature were obtained from cross-sectional studies related to the prevalence and frequency of obesity in older adults of Iran which had inclusion criteria. On the other hand, all other reviews, case-control, cohort, and intervention studies were excluded from the list of articles. In this study, for assessing gray literature (the documents and evidence not published), general searching was performed in Google database and other subject-related websites. Duplicate publication and multiple publications from the same population were removed using citation management software EndNote (version X7, for Windows, Thomson Reuters).

### Quality assessment

To check the quality of cross-sectional studies, STROBE checklist was used. This checklist included 22 sections of which, 18 were general and useful for all kinds of observational studies (such as cohort, case-control, and sectional). On the other hand, 4 were specific to the type of study, and included different aspects of methodology such as determining the appropriate sample size, type of study, sampling method, study population, data collection method, description of variables and sample assessment method, data collection tool, aims of the study, the statistical tests used, and statement of study results. We evaluated the items of the STROBE checklist that were adequately reported. The total number of items on the STROBE checklist is 32. Some items are specific for some study designs only (e.g. cohort or case control). Consequently, if an item was not applicable for the study design, it was scored as ‘not applicable’. For a more detailed description of the requirements to score ‘adequately reported’, Items were either scored as ‘adequately reported’ (score 1) or ‘not applicable’ (score 0). If any of these items were not applicable for the study design, it was scored as ‘not applicable’. ‘Not applicable’ items were not added to the amount of items to score. (The STROBE Statement was introduced to improve the quality of reporting of observational studies. The aim of the STROBE statement is to increase transparency in reporting. No studies have been excluded from the quality assessment.)

### Statistical analysis

The prevalence of obesity in older adults was obtained from each study. Heterogeneity of studies was assessed using *I*^2^ test. Generally, heterogeneity is categorized in 3 groups: heterogeneity less than 25% (low heterogeneity), between 25 and 75% (medium heterogeneity), and more than 75% (high heterogeneity). According to its results (*I*^2^ = 98%), and given the high heterogeneity in the included studies, the random effects model was used to combine the results of studies. The probability of publication bias in results was assessed using Egger test, as well as 0.05 significance level, plus Begg and Mazumdar’s rank correlation test and 0.1 significance level. The data were analyzed using Comprehensive Meta-analysis software (Biostat, Englewood, NJ, USA version 3).

## Results

### Search outcome

**Fig. 1:**
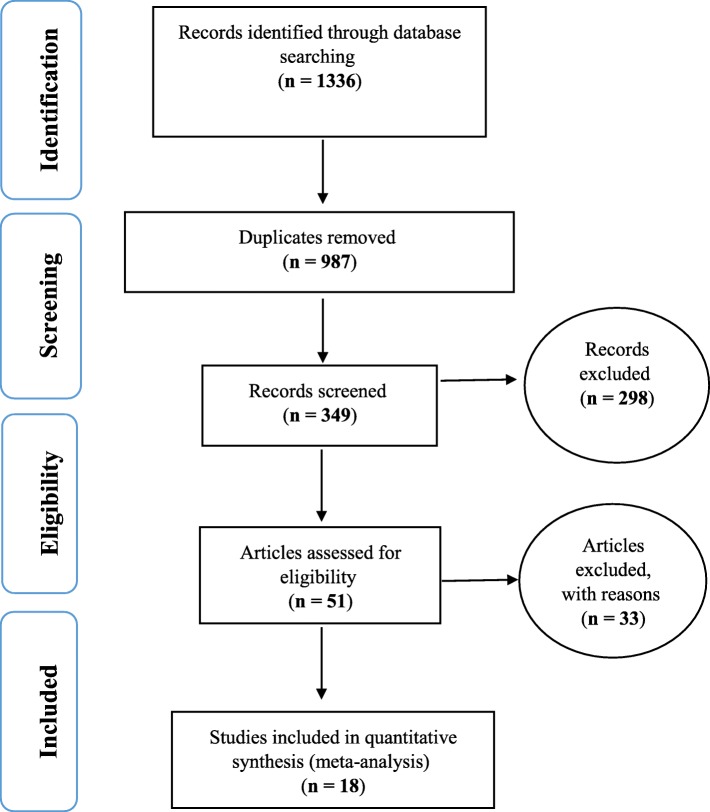
The flowchart on the stages of including the studies in the systematic review and meta-analysis (PRISMA 2009)

Based on the evaluation performed on prevalence of obesity in older adults of Iran and articles published in both the internal and international journals and searching in databases, 61 articles were obtained from SID and Magiran databases, 592 articles from Medline (PubMed), 434 articles from Science Direct, 113 articles from Scopus, and 136 articles from Google Scholar. Afterwards, from articles with primary inclusion criteria, 987 repeated articles were removed and 349 articles remained. Finally, 298 subject-unrelated articles, and 33 articles whose complete contents were not accessible were removed (In the search for articles in Iranian sources, because most of these articles were outdated, the original text of the articles was not available to researchers, in addition most studies in the secondary assessment did not mention prevalence and thus 33 articles were excluded at this stage). Eventually, 18 articles entered the meta-analysis and were prepared based on evaluating the quality of studied articles, according to PRISMA 2009 (Fig. 1).

Specifications of studies included in the study included the authors’ name, the title of the article, year and place the study had been performed, sample size, frequency, and prevalence of obesity among older adults studied [12–29] (Table 1).

**Table 1:** Specifications of studies included in the study

Row	Author	Publication year	Area	Participants’ Age	Sample size	Prevalence	Quality assessment
1	Aliabadi [12]	2007	Khorasan Razavi	70 ± 7.8	1962	11.3	Moderate
2	Ahmadi [13]	2000	Yasuj	> 55	109	33.1	Moderate
3	Zainali [14]	2014	Tehran	70.8 ± 7.1	368	19.3	High
4	Ostovar [15]	2017	Bushehr	67.9 ± 7.08	3000	24.1	High
5	Taghipour [16]	2014	Amol	57.4 ± 13.2	366	17.8	Moderate
6	Shamsi [17]	2012	Tehran	75.2 ± 7.9	310	14.5	Moderate
7	Hossaini [18]	2007	Babol (Amishahr)	70 ± 7	1019	44.2	Moderate
8	Mohammadi [19]	2016	Tehran	60–70	375	25	High
9	Ghorbani [20]	2000	Semnan	50–55	177	31.1	Moderate
10	Payman [21]	2011	Ilam	70.4 ± 11	121	16	Moderate
11	Mirzaei [22]	2017	Yazd	> 50	9211	14.1	High
12	Aghaalinejha d [23]	2013	Iran (Ardbil, Isfahan, Ahwaz, Tehran, Kerman, Mashhad)	> 50	261	26	High
13	Mahdavi [24]	2014	Kashan	72.07 ± 9.03	500	12.6	High
14	Khalili [25]	2014	Kashan	67.7 ± 6.8	400	18.8	Moderate
15	Alavi [26]	2006	Isfahan	> 60	1694	20.5	Moderate
16	Hosainipanah [27]	2009	Tehran	> 50	4402	36.8	Moderate
17	Nemati [28]	2009	Khorasan Razavi	> 60	1962	11.7	Moderate
18	Barzin [29]	2014	Tehran	> 60	3706	30.3	Moderate

### Publication bias

**Fig. 2:**
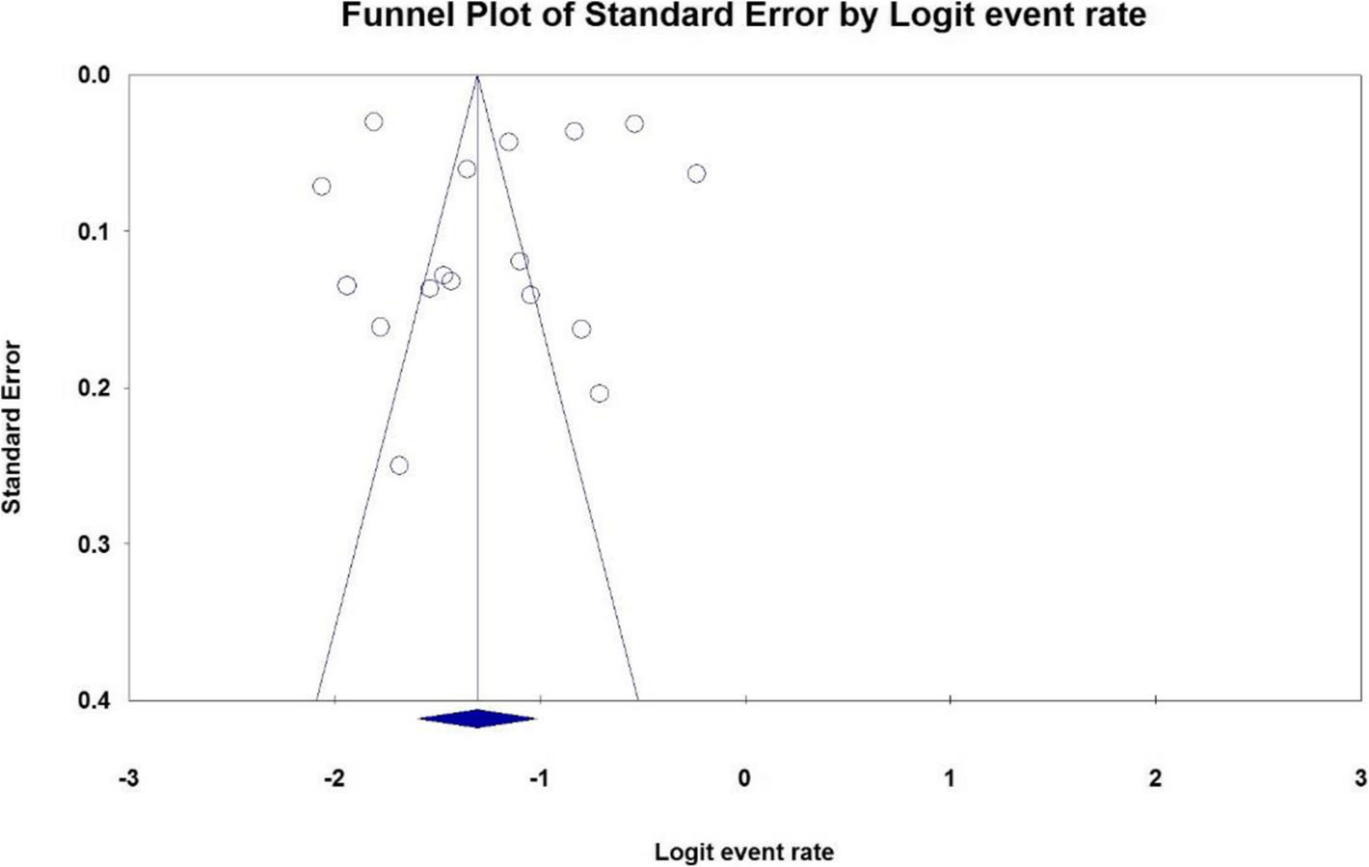
Funnel plot of results related to the prevalence of obesity in older adults of Iran

The probability of publication bias in results was assessed using funnel plot (Fig. 2), plus Egger test, which showed that publication bias was not statistically significant (*P* = 0.544). Further, regarding the large sample size, the analysis was performed using Begg and Mazumdar’s rank correlation test, which indicated that publication bias was not statistically significant (*P* = 0.969).

### Meta-analysis

**Fig. 3:**
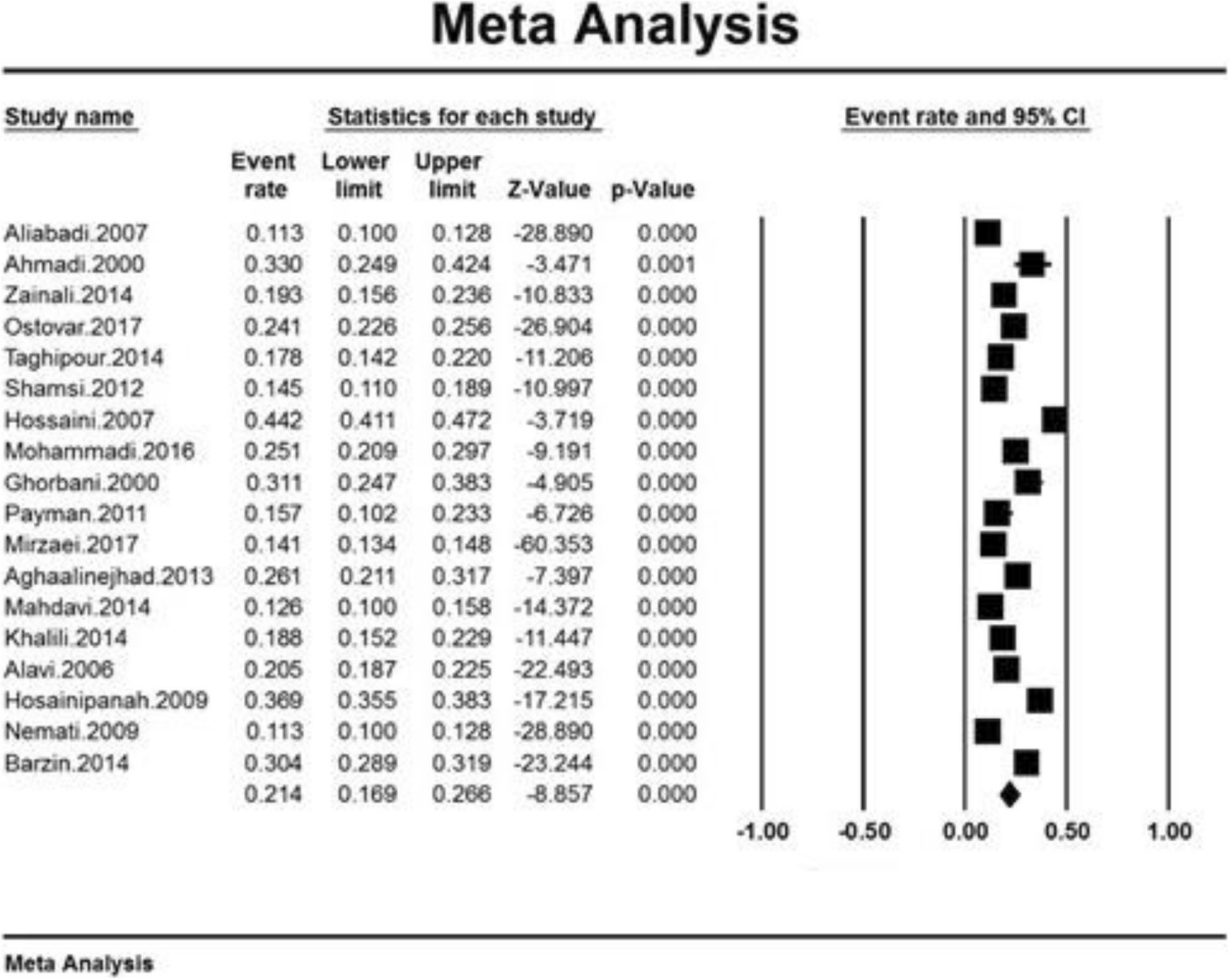
Total prevalence of obesity in older adults of Iran based on the random effects model

A total of 29,943 subjects participated. The total prevalence of obesity in older adults of Iran was obtained to be 21.4% (95% CI: 26.6–16.9%) based on the meta-analysis. The highest obesity prevalence obtained in older adults of Babol (Amir Shahr) was 44.2% (95% CI: 41.1–47.2%) in 2007 [18], while the minimum obesity prevalence obtained in older adults of Razavi Khorasan was 11.3% (95% CI: 10–12.8%) in 2007 [12] (Fig. 3). Fig. 3 shows the rate of obesity prevalence in Iranian older adults based on the random-effects model, where, black square represents the prevalence with the length of the line on which the square is placed showing 95% confidence Interval (CI) in each study. Finally, the diamond sign indicates the prevalence across the entire country for all studies.

### Sensitivity analysis

**Fig. 4:**
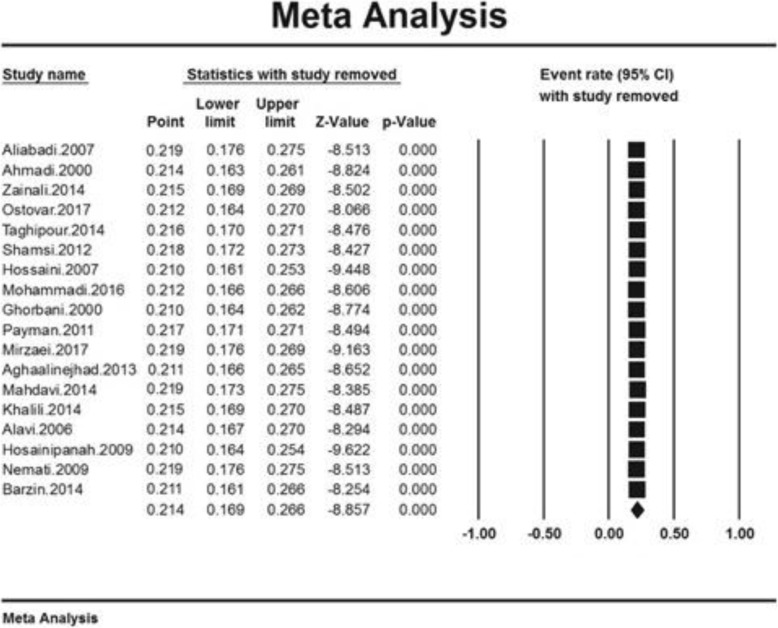
Results of sensitivity analysis

A sensitivity analysis was performed to ensure the stability of results, i.e. after removal, the study results did not change (Fig. 4).

### Sub-group analysis

Table 2 reports the results of the sub-group analysis according to the quality of studies, year of publication, and sample size (Table 2).

**Table 2:** The results of sub-group analyses

Variables		No. studies	Prevalence% (95% CI)	I^2^ (%)	*P* value	No. participants
Quality of studies	Moderate	12	22.3 (16.7–29.1%)	98.7	0.000	16,228
	High	6	19.6 (14.7–25.7%)	97.4	0.000	13,715
Year of publication	2000–2005	2	31.8 (26.7–37.5%)	–	–	286
	2006–2011	6	21.2 (12.4–33.8%)	99	0.000	11,160
	2012–2018	10	19.7 (15.3–25.2%)	98.2	0.000	18,497
Sample size	≤1500	7	19.8 (13.5–28.1)	99.5	0.000	25,937
	> 1500	11	22.4 (16.2–30.1)	96.2	0.000	4006

### Meta-regression analysis

**Fig. 5:**
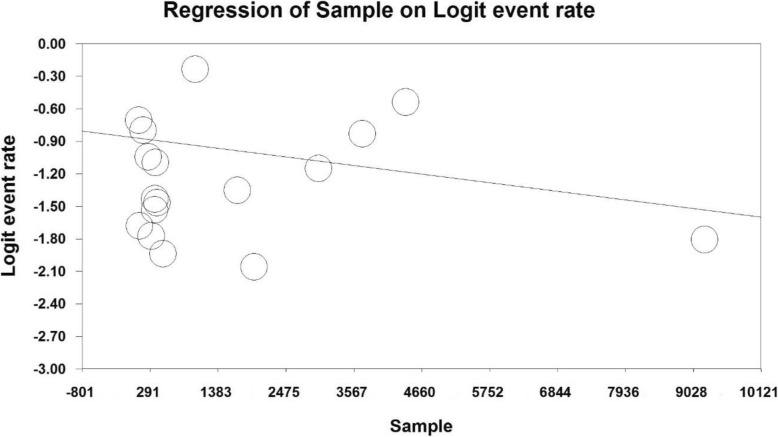
Meta-regression plot of the prevalence of obesity in older adults of Iran based on the sample size of the reviewed studies

**Fig. 6:**
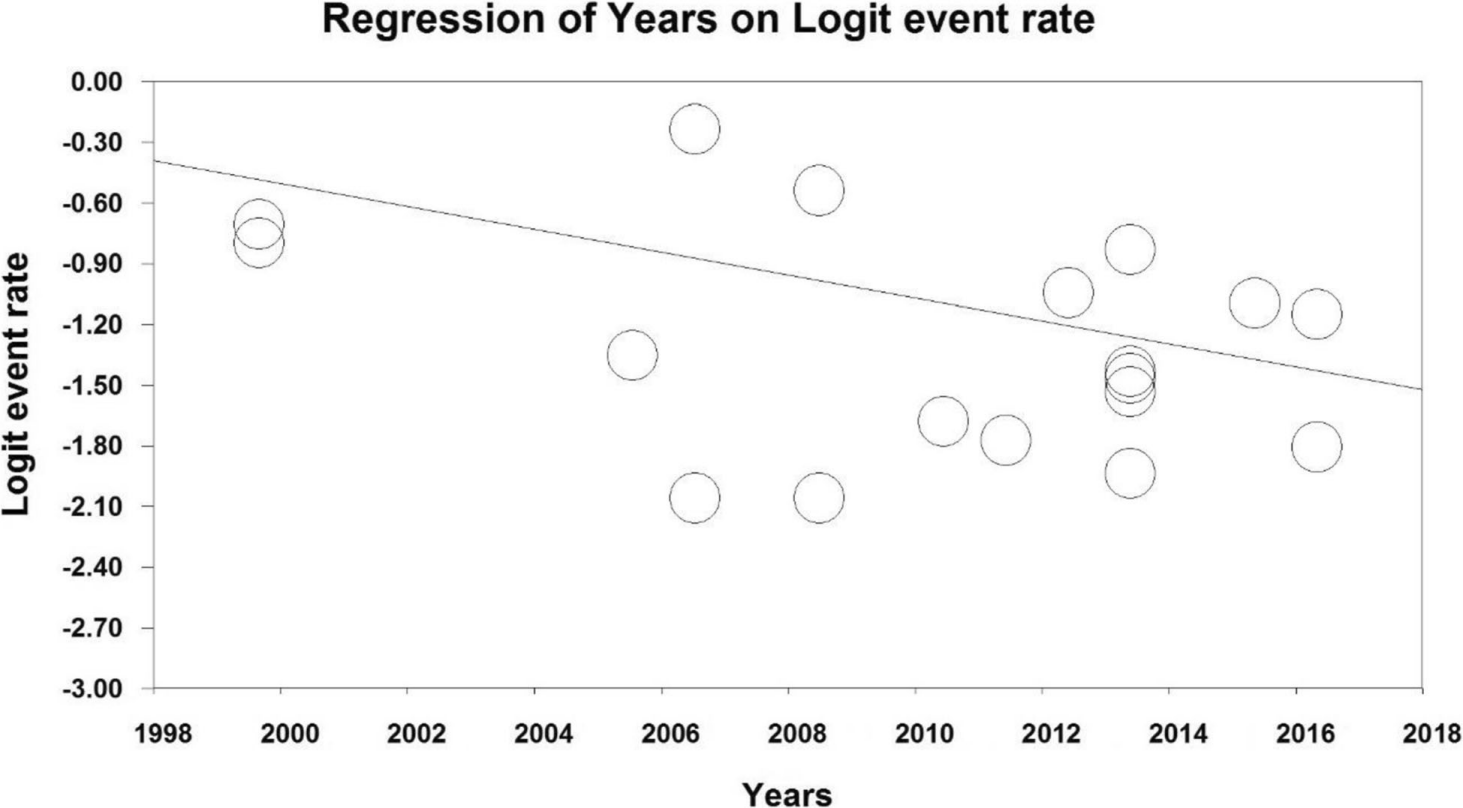
Meta-regression plot of the prevalence of obesity in older adults of Iran based on the publication year of the reviewed studies

For assessing the effects of potential factors affecting the heterogeneity of obesity prevalence in Iranian older adult people, meta-regression was employed for the sample size and study year factors (Figs. 5 and 6). Based on Fig. 5, as the sample size increased, obesity prevalence decreased in older adults of Iran (*p* < 0.05). Based on Fig. 6, as the study year increased, the obesity prevalence diminished in older adults of Iran (*p* < 0.05).

## Discussion

Older adults are highly susceptible to overweight and obesity due to reduced mobility [30]. Kuchak and colleagues [31], as well as Simoes and colleagues [32], reported that a lack of physical activity program and movement in older adults can intensify the growth of body mass and obesity. The results of this study indicated that total obesity prevalence based on the meta-analysis was 21.4. It was also found that as the sample size and study year increased, obesity prevalence decreased in older adults of Iran, with the differences being statistically significant. According to studies, 13% of adults were overweight [33]. The studies performed in USA showed that more than one-third of adults are overweight [34]. The study of Wee and colleagues [35] reported 18% obesity prevalence in older adults, indicating a higher prevalence of obesity in the older adults in Iran.

Another study performed in Spain showed that obesity prevalence was 22.9% in their adults and older adults [36]. Among Asian reports, obesity prevalence in older adults of Pakistan was 10.3% [37], and in Turkey was 15.6% [38], suggesting a higher prevalence of obesity in Iranian older adults than in the older adults of other Asian countries. On the other hand, the prevalence of obesity in older adults in Spain indicated a higher proportion compared to Iran.

The study performed in Saudi Arabia predicted that obesity prevalence in adult men will increase from 12% in 1992 to 41% in 2022, and in adult women will increase from 21 to 78%. This prevalence in women especially between 35 and 64 years old will highly increase which needs serious interventions [39], indicating a higher prevalence of obesity among older adults in Saudi Arabia compared to the older adult in Iran. This also highlights the importance of taking more serious measures in this country as well as the Eastern Mediterranean countries.

The study of Euronut-Seneca in Europe revealed that the obesity prevalence in elder women is 12 to 41%, and in elder men is 8 to 24% [40]. In different studies, again high prevalence was reported in females in comparison to males, where among older adults of Tunisia, the obesity rate in women was reported to be 21.7% and in men 9.4% [41]. In Spain, it was reported that obesity prevalence in women was 40.8%, and in men was 31.5% [42]. As stated earlier, the most important factor in obesity of older adults is lack of motivation and sedentary lifestyle. The most important difference given the higher obesity prevalence in elder women could be multiple births, hormonal differences, and physiologic, metabolic, as well as nutritional habit differences, concerning women’s attention to their physique and their fitness for beauty, and the consumption of more vegetables and the lack of consumption of fatty foods in them [43].

Different studies suggested that as the age in older adult year’s increases, the movement rate and physical activity of older adult decrease. In most studies, lack of movement was reported to be greater in elder women than in elder men and they noted that obesity in older adults can increase the risk of cardiovascular disease in them [44–46]. The study of Hosseini and colleagues [18] indicated that 76.3% of older adults did not practice regular walking. Meanwhile, a study performed in Britain showed that light and moderate physical activities such as regular walking, entertainment and sport activities such as swimming and slow running can significantly decrease obesity and overweight thus lowering the chance of incidence of cardiovascular disease [47]. As mentioned earlier, the most important factor affecting weight gain and obesity in older adults is their lack of movement which had also been mentioned in various studies.

### Limitation

The most important limitation of the present study was inaccessibility to the complete contents of the articles.

## Conclusion

According to the results of this study, the prevalence of obesity in the older adults of Iran is high. Regarding the growing rate of elderly population in Iran, health policymakers should pay more attention to exercise and movement of older adults and also prepare the required conditions for them to exercise sufficiently.

## Data Availability

Datasets are available through the corresponding author upon reasonable request.

## Abbreviations

BMI: Body Mass Index
PRISMA: Preferred reporting items for Systematic Reviews and Meta-Analysis.
SID: Scientific Information Database
STROBE: Strengthening the Reporting of Observational Studies in Epidemiology for cross- sectional Study
WHO: World Health Organization
